# Free water changes and their correlations with multimodal biomarkers in frontotemporal dementia

**DOI:** 10.1002/alz.71305

**Published:** 2026-03-22

**Authors:** Min Chu, Shaozhen Yan, Qianqian He, Ailing Yue, Yufei Chen, Jiahui Hou, William Robert Kwapong, Haitian Nan, Hong Ye, Pedro Rosa‐Neto, Miao Qu, Binbin Nie, Jie Lu, Liyong Wu

**Affiliations:** ^1^ Department of Neurology Xuanwu Hospital Capital Medical University Beijing China; ^2^ Department of Radiology and Nuclear Medicine Xuanwu Hospital, Capital Medical University Beijing China; ^3^ Alzheimer's Disease Research Unit McGill Centre for Studies in Aging Montréal, QuebeC Canada; ^4^ Beijing Engineering Research Center of Radiographic Techniques and Equipment Institute of High Energy Physics Chinese Academy of Sciences Beijing China; ^5^ School of Nuclear Science and Technology University of Chinese Academy of Sciences Beijing China; ^6^ National Clinical Research Center for Geriatric Diseases Beijing China

**Keywords:** free water, frontotemporal dementia, glymphatic system

## Abstract

**INTRODUCTION:**

The alterations of free water (FW) in behavioral variant frontotemporal dementia (bvFTD) and its clinical correlations remain unclear.

**METHODS:**

FW levels in the whole brain and specific white matter fiber tract was evaluated in 112 bvFTD patients and 103 normal controls.

**RESULTS:**

BvFTD patients exhibited elevated FW in the whole brain and regional white matter tracts, especially in the uncinate fasciculus and cingulum hippocampus. FW was correlated with perivascular spaces (PVSs) and diffusion tensor imaging analysis along the perivascular space (DTI‐ALPS) index. FW was also correlated with clinical scales, gray matter volume, and glucose metabolism. Additionally, FW was correlated with plasma glial fibrillary acidic protein and total tau. Mediation analyses showed that FW mediated the association between PVS and ALPS, and brain neurodegeneration mediated the association between FW and clinical scores.

**DISCUSSION:**

Elevated FW is involved in bvFTD and contributes to glymphatic dysfunction and neurodegeneration.

## Highlight

- FW correlates with glymphatic metrics (PVS, ALPS) and plasma GFAP in bvFTD.
- FW fully mediates the association between PVS and ALPS.
- GMV and cerebral metabolism mediate the association between FW and clinical severity/cognition in bvFTD.

## 1 BACKGROUND

Frontotemporal dementia (FTD) is a common form of early‐onset dementia, with the behavioral variant (bvFTD) representing its most frequent subtype.^1^ Pathologically, it is characterized by atrophy of the frontal and temporal lobes and clinically by behavioral symptoms.^1^ The glymphatic system plays a critical role in facilitating cerebrospinal fluid (CSF) flow through perivascular spaces (PVSs) to clear pathological proteins.^2^, ^3^, ^4^ Previous studies revealed impaired glymphatic function in bvFTD, reflected by a reduced diffusion tensor imaging analysis along the perivascular space (DTI‐ALPS) index, which correlates with clinical symptoms, brain changes, and peripheral biomarkers.^2^, ^5^ Furthermore, the efficiency of CSF‐interstitial fluid (ISF) exchange is another critical metric in the glymphatic system, which can be indicated by extracellular free water (FW).^6^, ^7^, ^8^ However, how extracellular FW changes in FTD patients remains unknown.

Impaired glymphatic function leads to the accumulation of waste products and fluid, resulting in elevated FW levels.^9^ It has been reported that FW is increased in various types of dementia, including Alzheimer's disease (AD)^9^, ^10^, ^11^, ^12^, ^13^, ^14^, ^15^, ^16^, ^17^, ^18^, ^19^ and dementia with Lewy bodies (DLB),^20^ and is associated with clinical features and pathological progression. In FTD, we hypothesize that impaired glymphatic activity expands the extracellular space and increases FW, which may further exacerbate other pathophysiological processes such as neurodegeneration, clinical severity, and neuroinflammation. Therefore, investigating FW alterations and their clinical correlations provides valuable insight into glymphatic dysfunction and the underlying pathophysiology of FTD.

This study systematically examines white matter FW (WM‐FW) changes in patients with bvFTD and analyzes their correlations with glymphatic metrics, including PVS and the ALPS index, clinical scales, brain volume, cerebral metabolism, and plasma biomarkers. Additionally, we aim to explore potential mediating mechanisms behind these associations. The findings are expected to support the clinical utility of FW as an imaging biomarker for bvFTD and provide new perspectives on the glymphatic function and pathophysiological mechanisms in FTD.

## 2 METHODS

### 2.1 Ethics

All participants were recruited between January 2022 and June 2025 from the Department of Neurology in Xuanwu Hospital, including both outpatients and inpatients, as well as healthy volunteers enrolled through community health screenings during the same period. Written informed consent was obtained from all individuals, and the study protocol was approved by the Institutional Medical Ethics Committee.

### 2.2 Participants

Between January 1, 2022, and June 1, 2025, a cohort of right‐handed participants was recruited from the Department of Neurology at Xuanwu Hospital. The study included 112 patients diagnosed with behavioral variant frontotemporal dementia (bvFTD) and 103 normal controls (NC) matched for age, sex, and years of education. Participant matching was implemented to enhance statistical robustness and reduce potential confounding variables. All bvFTD diagnoses were consistent with the 2011 international diagnostic criteria for probable bvFTD.^21^ Healthy controls exhibited no evidence of cognitive impairment, depression, or anxiety and performed within normal ranges on neuropsychological assessments, as indicated by Mini‐Mental State Examination (MMSE) scores ≥ 24 and a Clinical Dementia Rating (CDR) plus National Alzheimer's Coordinating Center (NACC) frontotemporal lobar degeneration (FTLD) score of 0 in both behavior/comportment and language domains.

Exclusion criteria applied to all subjects were as follows: (1) major neurological or psychiatric disorders that could affect cognitive performance, including substance use disorder, alcohol dependence, schizophrenia, intracranial neoplasms, or cerebrovascular disease; (2) standard contraindications to magnetic resonance imaging (MRI); and (3) absence of a reliable informant for clinical history gathering. Neither group included individuals with hypertension, hypercholesterolemia, diabetes, smoking history, or prior vascular events. All participants had a body mass index (BMI) within the normal range (18.5 to 23.9 kg/m^2^).

### 2.3 Clinical assessments

All participants underwent a standardized series of neuropsychological evaluations. Global cognitive function was screened using the Mini‐Mental State Examination (MMSE) and the Montreal Cognitive Assessment (MoCA), while disease severity was evaluated with the CDR^®^ plus NACC FTLD. Behavioral symptom severity was measured using the Frontal Behavior Inventory (FBI), which comprises two subscales: the negative apathy subscale (items 1 to 12) and the positive disinhibition subscale (items 13 to 24). Functional independence in daily life was assessed via the activities of daily living (ADL) scale.

### 2.4 Neuroimaging data acquisition

A hybrid 3.0T time‐of‐flight fluorodeoxyglucose F18‐positron emission tomography/MRI (^18^F‐FDG‐PET/MRI) scanner (SIGNA FDG‐PET/MR, GE Healthcare, WI, USA) equipped with a 19‐channel head and neck union coil was used to simultaneously capture MRI and FDG‐PET data. After injecting 3.7 MBq/kg of ^18^F‐FDG, we obtained both three‐dimensional (3D) T1‐weighted sagittal images and FDG‐PET volumes in a single session. The study involved acquiring various imaging data types characterized by specific parameters for further analysis as follows:

Acquisition parameters for T1‐weighted imaging data were as follows: repetition time (TR) = 6.9 ms, echo time (TE) = 2.98 ms, flip angle = 12°, inversion time = 450 ms, matrix dimensions = 256 × 256, field of view (FOV) = 256 × 256 mm^2^, slice thickness = 1 mm (192 sagittal slices, no inter‐slice gap), voxel size = 1 × 1 × 1 mm^3^, and total scan duration = 4 min 48 s.

Diffusion tensor imaging (DTI) data were acquired using a spin‐echo echo‐planar imaging (SE‐EPI) sequence: TR = 16,500 ms, TE = 97.6 ms, *b* value = 1000 s/mm^2^ (with diffusion gradients applied across 30 directions), 70 axial slices (no inter‐slice gap), FOV = 220 × 220 mm^2^, matrix size = 112 × 112, slice thickness = 2 mm, and number of excitations = 1.

Static ^18^F‐FDG‐PET data were collected with the following specifications: matrix size = 192 × 192, FOV = 350 × 350 mm^2^, pixel size = 1.82 × 1.82 × 2.78 mm^3^. Corrections were implemented for random coincidences, dead time, scatter, and photon attenuation; attenuation correction was performed via atlas‐based co‐registration of two‐point Dixon brain MRI. The default attenuation correction sequence (LAVA‐Flex, GE Healthcare) was acquired axially with TR = 4 ms, TE = 1.7 ms, and slice thickness = 5.2 mm (with 2.6 mm overlap) and was set automatically.

### 2.5 Neuroimaging data processing

**FIGURE 1 alz71305-fig-0001:**
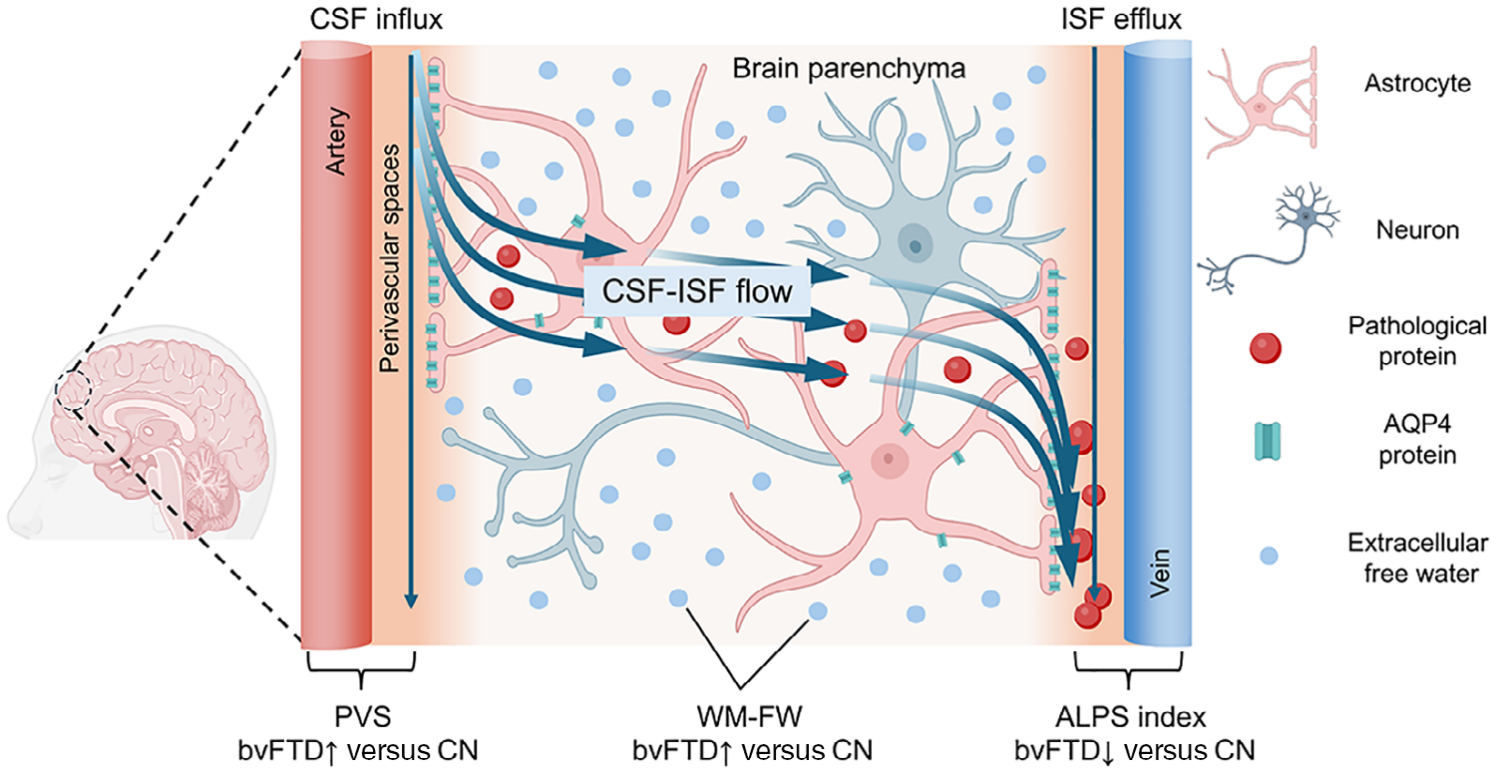
Schematic of cerebrospinal fluid (CSF)‐interstitial fluid (ISF) flow and solute clearance pathways of the glymphatic system in the brain. CSF infuses into the brain via the perivascular space (PVS), then circulates as CSF‐ISF flow through the brain parenchyma. The blue circles represent white matter‐free water (WM‐FW), and orange circles represent solute clearance in the brain parenchyma. ISF efflux occurs via the PVS at the diffusion tensor imaging analysis along the PVS (DTI‐ALPS) index. The bottom labels indicate representative magnetic resonance imaging biomarkers (e.g., PVS, WM‐FW, ALPS index), reflecting CSF‐ISF dynamics and solute transport efficiency.

Image processing followed standardized pipelines: glymphatic function‐related MRI markers, including FW, PVS, and ALPS (Figure 1).

### 2.6 PVS volume quantification

PVS burden was quantified using an automated pipeline implemented in Quantitative Imaging Toolkit (QIT) and FMRIB software library (FSL). First, FreeSurfer‐derived segmentation outputs (brain.mgz containing intracranial tissues of T1WI image and aseg.mgz containing anatomical labels) were converted to NIfTI format and renamed brain_fs.nii.gz and aseg_fs.nii.gz, respectively. Then, a whole‐brain mask was generated from the aseg_fs.nii.gz file in FSL. To enhance the detection of small tubular structures, the T1WI images were first denoised using a non‐local means (NLM) filter (via QIT's VolumeFilterNLM). Subsequently, a Frangi vesselness filter (via QIT's VolumeFilterFrangi) was applied to the denoised images to enhance tubular structures. The filter computes Hessian matrix eigenvalues to quantify “vesselness,” configured with the following parameters: lower scale range limit set to 0.1 and dark object mode enabled. The resulting vesselness map was normalized to a standard score (z‐score) by subtracting the mean and dividing by the standard deviation of the whole‐brain voxel intensities using FSL fslmaths. To generate the final binary PVS mask, a threshold of 2.3 was applied to the standardized vesselness map (i.e., voxels with a z‐score > 2.3 were retained as PVS). Finally, the total PVS volume (mm^3^) was calculated by summing the voxel volumes in the binary mask.

**RESEARCH IN CONTEXT**

1. **Systematic review**: We evaluated existing literature via PubMed and key conference abstracts, identifying as FW emerging but understudied mechanisms in behavioral variant frontotemporal dementia (bvFTD). Prior studies mainly explored FW in AD, with little research effort devoted to its alterations and correlations in bvFTD.
2. **Interpretation**: Our findings reveal a central role of elevated FW in bvFTD pathophysiology, establishing its links with glymphatic dysfunction, neurodegeneration, inflammation, and clinical severity. We demonstrate FW contributions to glymphatic function and clinical manifestations.
3. **Future directions**: There are key questions regarding (1) the temporal sequence of FW alterations, (2) potential therapeutic strategies targeting FW, and (3) generalizability to other neurodegenerative disorders.

### 2.7 DTI data preprocessing (for FW and ALPS)

DTI was preprocessed using a combination of MRtrix3 and FSL tools. First, 4D DICOM data were converted to NIfTI and denoised using the MP‐PCA algorithm (dwidenoise, MRtrix3), followed by Gibbs ringing artifact correction (mrdegibbs, MRtrix3). To correct for susceptibility‐induced distortions, eddy currents, and motion, we employed FSL's topup and eddy tools. The b0 images acquired in both anterior‐posterior (AP) and posterior‐anterior (PA) phase‐encoding directions were used as input for topup to estimate the susceptibility field. This fieldmap was then fed into eddy to apply the corrections to the full diffusion dataset.

### 2.8 FW estimation

The FW fraction maps[Fig alz71305-fig-0001] were generated by fitting a regularized bi‐tensor model to the preprocessed DTI images, implemented in Dipy (Diffusion Imaging in Python, version 1.5.0; https://dipy.org/). The model accounts for the presence of both tissue and FW compartments within each voxel, comprising restricted tissue water and FW, as shown in Equation (1):

(1) $$S_{i}=S_{o}\left[\left(1-F_{w}\right)\cdot e{-}^{bg_{i}^{T}Dg_{i}}+F_{w}\cdot e^{-bD_{w}}\right],$$

where *S_0_* stands for the b0 image, *S_i_* stands for the *i*th gradient direction image, *Dw* stands for the isotropic diffusion coefficient of FW, fixed at 3.0×10^−3^mm^2^/s, and *Fw* stands for the proportion of FW in each voxel.

To stabilize parameter estimation and avoid non‐physiological solutions, we employedregularization constraints during optimization, comprising FA > 0.7, which reflects typical maximum FW content in WM, and MD > 2.5, which reflects typical maximum FW content in CSF. For each voxel, the optimization converged when the relative change in residuals fell below 10^−6^ or after 100 iterations. The resulting Fw values were mapped to generate voxel‐wise FW fraction maps, with units expressed as a dimensionless fraction (0‐1). This approach leverages Dipy's robust implementation of the regularized bi‐tensor framework, enabling accurate separation of FW from restricted tissue diffusion even with single‐shell DTI data, while mitigating overfitting through physiologically constrained regularization.

### 2.9 ALPS index computation

Fractional anisotropy (FA) and directional diffusivity maps (x, y, z axes) were generated from the preprocessed DTI data using FSL's dtifit. Each subject's FA map was non‐linearly registered to the JHU‐ICBM‐FA template, and the resulting transformation was applied to the diffusivity maps for spatial normalization. Three cubic ROIs (3 mm isotropic) were placed bilaterally in the superior corona radiata (SCR, projection fibers) and superior longitudinal fasciculus (SLF, association fibers) at the level of the lateral ventricle body, guided by the JHU DTI atlas. ROI positions were independently verified by two experienced researchers (MC and BBN). Diffusivity values (Dxx, Dyy, Dzz) were extracted from these ROIs. The ALPS index was calculated as the mean of bilateral values using the formula

$$ALPS\;index=\frac{mean(D_{xxproj},D_{xxassoc})}{mean(D_{yyproj},D_{zzassoc})}$$

MRI and ^18^F‐FDG‐PET: (1) T1: DICOM images were converted to NIfTI format using MRIcron for gray matter volume (GMV) estimation, with voxel‐based morphometry (VBM) conducted using the CAT12 toolbox in SPM12 with default settings and the “East Asian Brain” ICBM template, T1 images segmented into gray matter, white matter, and CSF, DARTEL registration employed for high‐dimensional normalization to MNI space, and smoothed images obtained with an 8 mm FWHM Gaussian kernel; (2) FDG‐PET images: preprocessed in SPM12 running under MATLAB, with spatial normalization parameters derived from T1 images applied to co‐registered FDG‐PET data, images smoothed with an 8‐mm FWHM kernel and intensity‐normalized to the cerebellar region to produce standardized uptake value ratio (SUVR) maps; and ROI‐based analysis utilized the Automated Anatomical Labeling (AAL) atlas comprising 90 brain regions to extract regional GMV and SUVR values.

### 2.10 Plasma biomarker measurements

Fasting blood samples were collected in EDTA‐containing tubes (BD, USA) and centrifuged at 3000 rpm for 10 min to isolate plasma, which was aliquoted and stored at −80°C until analysis. Biomarker quantification included S‐PLEX electrochemiluminescence immunoassay for measuring glial fibrillary acidic protein (GFAP), neurofilament light chain (NFL), and *t*‐tau levels using Kit K15639S‐X according to the manufacturer's instructions; ELISA for assessing transforming growth factor‐beta 1 (TGF‐β1), vascular endothelial growth factor A (VEGFA), and C‐X3‐C motif chemokine ligand 1 (CX3CL1) following kit protocols; and Luminex xMAP multiplex assays for measuring matrix metalloproteinase‐9 (MMP‐9), platelet‐derived growth factor‐BB (PDGF‐BB), galectin‐3, and vascular cell adhesion molecule‐1 (VCAM‐1) with samples incubated with antibody‐conjugated magnetic beads, followed by the addition of biotinylated detection antibodies and streptavidin–phycoerythrin, data acquired on a Luminex X‐200 system, and concentrations determined using Milliplex Analyst software (version 5.1), while quality controls and batch processing were implemented to minimize technical variability. Out of the total cohort of 112 patients with bvFTD, all patients completed clinical assessments and MRI data collection. However, plasma biospecimens were only available for a subset of 49 patients, and all biomarkers were successfully measured.

### 2.11 Statistical analysis

Data were analyzed using Python 3.11.7 and R 4.0.5 with a two‐tailed significance threshold of *p* < 0.05 adopted for all tests. Group differences in categorical variables such as sex were assessed using χ^2^ tests. Continuous variables were compared using independent *t*‐tests or Mann–Whitney U tests, depending on the data distribution. For group comparisons of FW within specific WM tracts, we applied false discovery rate (FDR) correction while adjusting for age, sex, and education. Partial correlation analyses controlling for age, sex, and education were conducted with FDR correction for multiple comparisons. Mediation analyses were performed using the MEDIATION package in R, with significance of indirect effects evaluated using bootstrap resampling with 5000 iterations.

## 3 RESULTS

### 3.1 Demographic and clinical indicators of participants

There was no significant difference in age, sex or education between the bvFTD and NC groups. The mean age at onset in the bvFTD group was 59.64 ± 6.27 years old, with a mean disease duration of 2.36 ± 1.54 years.

Cognitive assessment revealed markedly lower scores in the bvFTD group on both the MMSE (13.92 ± 7.36 vs 28.77 ± 1.26, *p* < 0.0001) and MoCA (9.07 ± 6.16 vs 26.15 ± 2.15, *p* < 0.0001). Activities of daily living were significantly impaired in bvFTD patients, as reflected by higher ADL scores (35.69 ± 14.25 vs 20 ± 0.00, *p* < 0.0001). Neurobehavioral symptoms were prominent, with elevated Neuropsychiatric Inventory Questionnaire scores (NPI‐Q) (26.37 ± 17.97) and total FBI scores (25.88 ± 12.93). Dementia severity measures also indicated significant impairment, with CDR Sum of Boxes (SOB) and FTLD‐CDR SOB scores of 7.44 ± 4.44 and 10.79 ± 4.88, respectively. The detailed demographics and clinical data are shown in Table 1.

**TABLE 1:** Demographics and clinical scales of participants.

	bvFTD	NC	*P*
*n*	112	103	NA
Sex (F/M)	54/58	49/54	0.9250
Age	62.05 ± 6.39	60.66 ± 7.65	0.1474
Age at onset	59.64 ± 6.268	NA	NA
Education	8.92 ± 4.28	9.64 ± 2.09	0.1185
Disease course	2.36 ± 1.54	NA	NA
Behavior	2.29 ± 0.81	0	<0.0001
Language	1.06 ± 0.88	0	<0.0001
CDR SOB	7.44 ± 4.44	0	<0.0001
CDR® plus NACC FTLD‐SOB	10.79 ± 4.88	0	<0.0001
MMSE	13.92 ± 7.36	28.77 ± 1.26	<0.0001
MoCA	9.07 ± 6.16	26.15 ± 2.15	<0.0001
ADL	35.69 ± 14.25	20 ± 0	<0.0001
NPI‐Q	26.37 ± 17.97	0	<0.0001
FBI	25.88 ± 12.93	0	<0.0001
FBI‐apathy	18.64 ± 8.33	0	<0.0001
FBI‐disinhibition	7.24 ± 6.07	0	<0.0001

Abbreviations: ADL, activities of daily living scale; bvFTD, behavioral variant frontotemporal dementia; CDR SOB, the sum of the boxes scores of the 6 domains of the CDR; CDR® plus NACC FTLD‐SOB, the sum of the boxes scores of the 6 domains of the CDR plus the behavior and language domains; FBI, Frontal Behavioral Inventory; FDG, Fluorodeoxyglucose Uptake; GMV, gray matter volume; MMSE, Mini‐Mental State Examination; MoCA, Montreal cognitive assessment; NC, normal control; NPI‐Q, Neuropsychiatric Inventory Questionnaire.

### 3.2 Alterations of WM‐FW in bvFTD group

**FIGURE 2:**
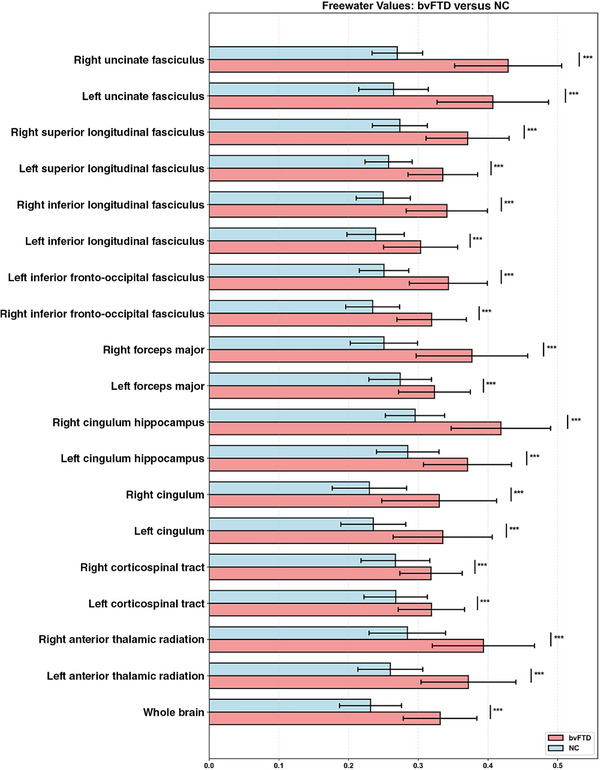
Free water (FW) alterations in patients with bvFTD. This figure presents the results of group comparison of FW measures between patients with behavioral variant frontotemporal dementia (bvFTD) and normal controls (NCs). The measures include whole‐brain FW levels and FW levels of specific white matter tracts (bilateral anterior thalamic radiation, corticospinal tract, cingulum, cingulum hippocampus, forceps major, inferior fronto‐occipital fasciculus, inferior longitudinal fasciculus, superior longitudinal fasciculus, uncinate fasciculus). The comparison indicators and significance are displayed in the figure. Detailed mean and standard deviation are presented in Table 2. ***p < 0.001, **p < 0.01, *p < 0.05.

Significant differences in FW were observed in all regions examined[Fig alz71305-fig-0001] (Figure 2 and Table 2). The bvFTD group exhibited consistently higher FW values compared to the NC group across both whole‐brain and regional WM tracts (all *p* < 0.0001). Globally, the whole‐brain FW value was significantly elevated in bvFTD patients (0.3313 ± 0.0528) relative to controls (0.2316 ± 0.0444). Particularly pronounced differences were in the uncinate fasciculus (left: 0.4069 ± 0.0799 vs 0.2645 ± 0.0499; right: 0.4290 ± 0.0768 vs 0.2699 ± 0.0364) and the hippocampus of the cingulum (right: 0.4185 ± 0.0713 vs 0.2954 ± 0.0425).

**TABLE 2:** Group comparison of free water levels between bvFTD and NC group.

Region	bvFTD	NC	*P*	FDR‐corrected *p*
Whole brain	0.33 ± 0.05	0.23 ± 0.04	4.03 × 10^−^^3^ ^4^	1.91 × 10^−^^3^ ^3^
Left anterior thalamic radiation	0.37 ± 0.07	0.26 ± 0.05	1.14 × 10^−^^3^ ^1^	3.60 × 10^−^^3^ ^1^
Right anterior thalamic Radiation	0.39 ± 0.07	0.28 ± 0.05	5.79 × 10^−^^2^ ^6^	9.17 × 10^−^^2^ ^6^
Left corticospinal tract	0.32 ± 0.05	0.27 ± 0.05	2.38 × 10^−^^1^ ^3^	2.66 × 10^−^^1^ ^3^
Right corticospinal tract	0.32 ± 0.04	0.27 ± 0.05	7.40 × 10^−^^1^ ^3^	7.81 × 10^−^^1^ ^3^
Left cingulum	0.34 ± 0.07	0.24 ± 0.05	5.30 × 10^−^^2^ ^5^	7.75 × 10^−^^2^ ^5^
Right cingulum	0.33 ± 0.08	0.23 ± 0.05	7.15 × 10^−^^2^ ^0^	9.06 × 10^−^^2^ ^0^
Left cingulum hippocampus	0.37 ± 0.06	0.28 ± 0.04	3.03 × 10^−^^2^ ^4^	4.11 × 10^−^^2^ ^4^
Right cingulum hippocampus	0.42 ± 0.07	0.30 ± 0.04	1.95 × 10^−^^3^ ^6^	1.86 × 10^−^^3^ ^5^
Left forceps major	0.32 ± 0.05	0.27 ± 0.04	1.12 × 10^−^^1^ ^2^	1.12 × 10^−^^1^ ^2^
Right forceps major	0.38 ± 0.08	0.25 ± 0.05	1.72 × 10^−^^3^ ^0^	3.64 × 10^−^^3^ ^0^
Right inferior fronto‐occipital fasciculus	0.32 ± 0.05	0.23 ± 0.04	5.05 × 10^−^^3^ ^1^	1.20 × 10^−^^3^ ^0^
Left inferior fronto‐occipital fasciculus	0.34 ± 0.06	0.25 ± 0.04	8.55 × 10^−^^3^ ^3^	3.25 × 10^−^^3^ ^2^
Left inferior Longitudinal fasciculus	0.30 ± 0.05	0.24 ± 0.04	2.59 × 10^−^^1^ ^9^	3.08 × 10^−^^1^ ^9^
Right inferior longitudinal fasciculus	0.34 ± 0.06	0.25 ± 0.04	4.58 × 10^−^^3^ ^0^	7.92 × 10^−^^3^ ^0^
Left superior longitudinal fasciculus	0.34 ± 0.05	0.26 ± 0.03	3.12 × 10^−^^3^ ^0^	5.93 × 10^−^^3^ ^0^
Right superior longitudinal fasciculus	0.37 ± 0.06	0.27 ± 0.04	2.15 × 10^−^^3^ ^1^	5.84 × 10^−^^3^ ^1^
Left uncinate fasciculus	0.41 ± 0.08	0.26 ± 0.05	1.36 × 10^−^^3^ ^5^	8.63 × 10^−^^3^ ^5^
Right uncinate Fasciculus	0.43 ± 0.08	0.27 ± 0.04	5.36 × 10^−^^4^ ^7^	1.02 × 10^−^^4^ ^5^

Adjusted for age, sex, and education.

Abbreviations: bvFTD, behavioral variant frontotemporal dementia; NC, normal control.

### 3.3 Correlation between FW and other glymphatic metrics

**FIGURE 3:**
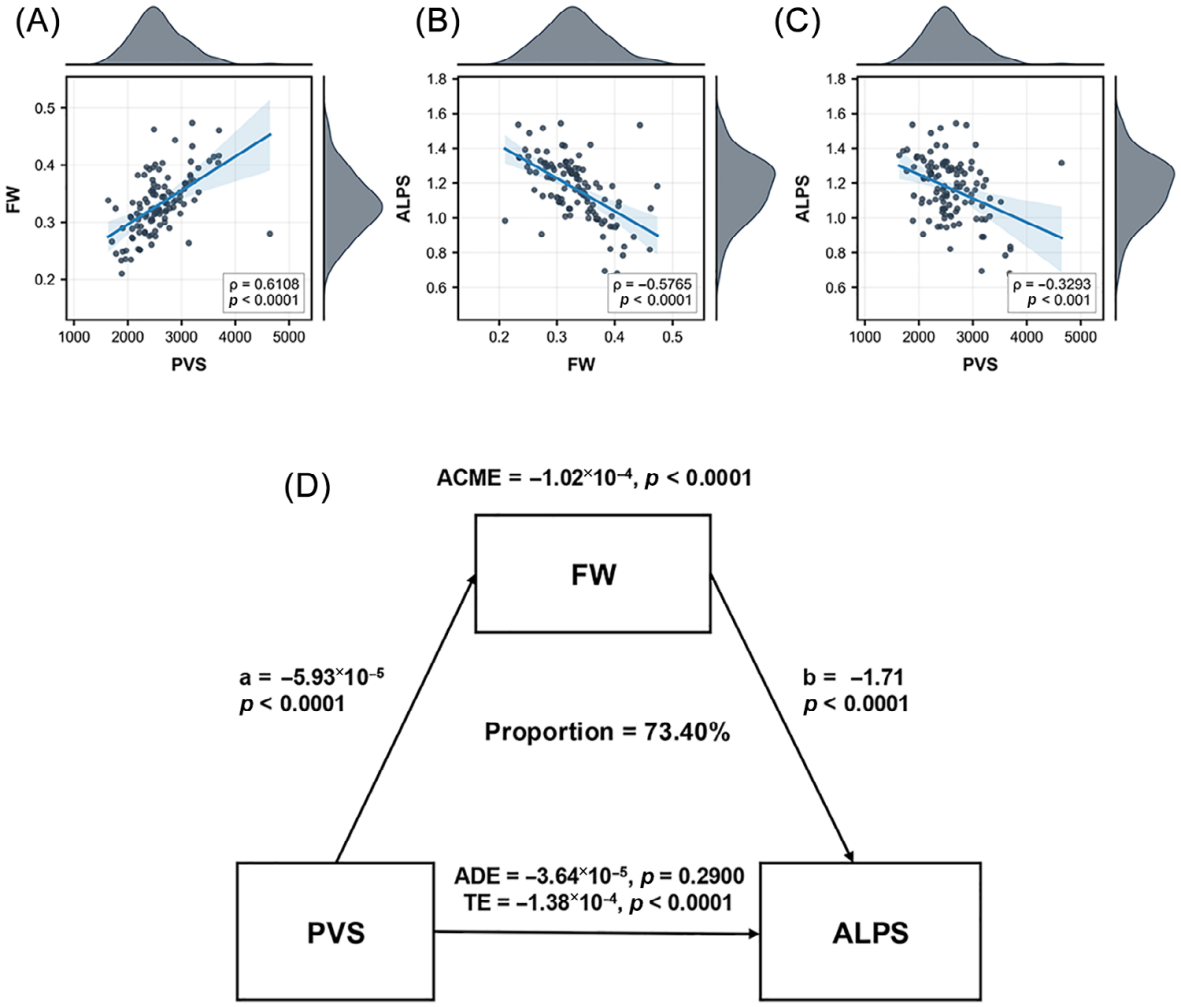
Correlation and mediation analysis of PVS, FW, and ALPS. Scatter plots showing correlation between variables. (A) Positive correlation between PVS volume and FW (*R* = 0.6108, *p* < 0.0001). (B) Negative correlation between FW and diffusion tensor imaging analysis along perivascular spaces (DTI‐ALPS) (*R* = −0.5765, *p* < 0.0001). (C) Negative correlation between PVS and DTI‐ALPS (*R* = −0.3293, *p* < 0.0010). Shaded areas represent 95% confidence intervals. (D) Mediation model illustrating pathway among PVS, FW, and ALPS. The model explained 73.4% of the variance in ALPS. The indirect effect of PVS on ALPS via FW was significant (ACME = −1.02×10^−^^4^, *p* < 0.0001), while the direct effect of PVS on ALPS was non‐significant after accounting for mediation (ADE = −3.64 × 10^−5^, *p *= 0.2900). ACME, average causal mediation effect; ADE, average direct effect; FW, free water; DTI‐ALPS, diffusion tensor imaging analysis along the perivascular space; PVS, perivascular space volume.

To characterize the relationships among the glymphatic variables, we first conducted partial correlation analyses in the bvFTD group (Figure 3A–C). A positive correlation was observed between PVS and FW (*r *= 0.6168, *p* < 0.0001), while FW was negatively correlated with ALPS (*r *= −0.5756, *p* < 0.0001), and ALPS was negatively correlated with PVS (*r *= −0.3293, *p* < 0.0010). We then constructed a mediation model (Figure 3D) to explore the underlying pathway linking PVS, FW, and ALPS. The indirect effect of PVS on ALPS via FW was significant (average causal mediation effect [ACME] = −1.02×10^−4^, *p* < 0.0001), whereas the direct effect of PVS on ALPS was not (ADE = −3.64×10^−5^, *p* = 0.2900).

### 3.4 Correlation between FW and clinical scales, neuroimages, and plasma biomarkers

**FIGURE 4:**
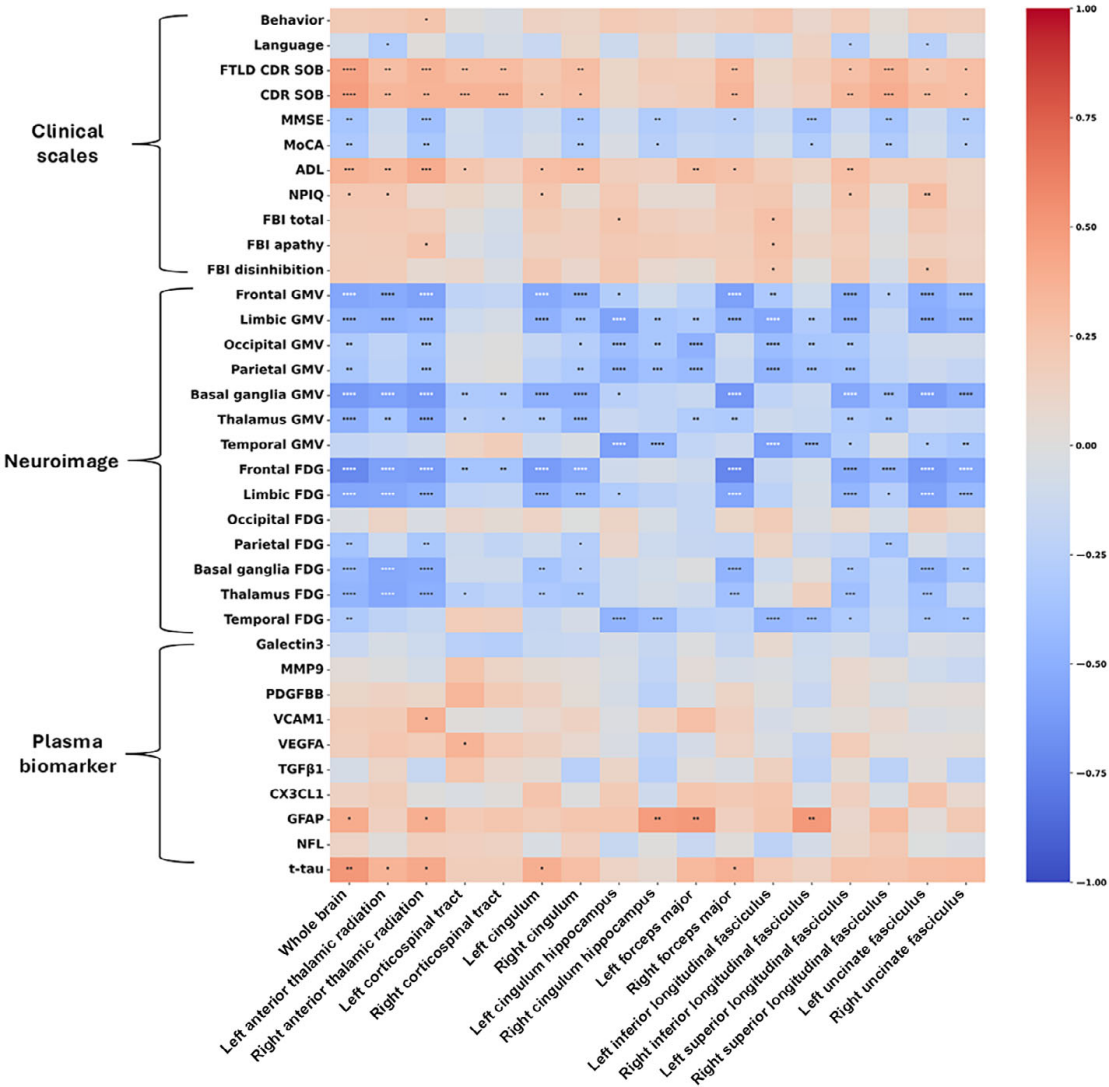
Heatmap of partial correlation analysis between FW and clinical scales, neuroimages, and plasma biomarkers. This heatmap shows the results of partial correlation analysis between cerebral FW levels and clinical scale scores, neuroimaging indicators, and plasma biomarkers in patients with bvFTD. The color intensity in the heatmap reflects the strength and direction of the correlation between each indicator (red values indicate a positive correlation, blue values indicate a negative correlation; the darker the color and the larger the absolute value of the numerical value, the stronger the correlation). Confounding factors such as age, sex, and education level were controlled during the analysis. The statistical significance threshold used for the correlation analyses is *p* < 0.05 before false discovery rate correction. ****p < 0.0001, ***p < 0.001, **p < 0.01, *p < 0.05. ADL, activities of daily living; GMV, gray matter volume; CDR SOB, Clinical Dementia Rating Scale Sum of Boxes; FTLD CDR SOB, the same as CDR plus NACC FTLD‐SB, sum of boxes scores of the six domains of the CDR plus the behavior and language domains; CX3CL1, C‐X3‐C motif chemokine ligand 1; FDG, fluorodeoxyglucose; FBI, frontal behavioral inventory; FBI apathy, frontal behavioral inventory–Apathy subscore; FBI disinhibition, frontal behavioral inventory–Disinhibition subscore; GFAP, glial fibrillary acidic protein; MMP9, Matrix Metallopeptidase 9; MMSE, Mini‐Mental State Examination; MoCA, Montreal Cognitive Assessment; NFL, neurofilament light chain; NPI‐Q, Neuropsychiatric Inventory Questionnaire; PDGF‐BB, platelet‐derived growth factor BB; TGF‐β1, transforming growth factor beta 1; *t*‐tau, total tau protein; VCAM1, vascular cell adhesion molecule 1; VEGFA, vascular endothelial growth factor A.

The results of partial correlation analyses examining the association between FW values and clinical, neuroimaging, and fluid biomarker variables are summarized in Figure 4 and Table 3.

**TABLE 3:** Partial correlation analysis between free water in whole brain and clinical scales, neuroimaging measures, and plasma biomarkers.

	Y	R	*P*	FDR‐corrected *p*
Clinical	Behavior	0.2014	0.0357	0.0862
	Language	−0.0808	0.4038	0.4807
	CDR plus NACC FTLD‐SOB	0.4478	<0.0001	<0.0001
	CDR SOB	0.4741	<0.0001	<0.0001
	MMSE	−0.3446	0.0002	0.003
	MoCA	−0.3133	0.0009	0.0077
	ADL	0.3577	0.0001	0.0023
	NPI‐Q	0.2343	0.0142	0.0442
	FBI total	0.201	0.0361	0.0862
	FBI apathy	0.1747	0.0692	0.1233
	FBI disinhibition	0.1916	0.046	0.0966
Neuroimaging	Frontal GMV	−0.5493	<0.0001	<0.0001
	Limbic GMV	−0.4639	<0.0001	<0.0001
	Occipital GMV	−0.3073	0.0012	0.0028
	Parietal GMV	−0.3376	0.0003	0.001
	Basal ganglia GMV	−0.6247	<0.0001	<0.0001
	Thalamus GMV	−0.4777	<0.0001	<0.0001
	Temporal GMV	−0.1766	0.0662	0.1075
	Frontal FDG	−0.708	<0.0001	<0.0001
	Limbic FDG	−0.5507	<0.0001	<0.0001
	Occipital FDG	−0.0344	0.7407	0.7741
	Parietal FDG	−0.3549	0.0004	0.0012
	Basal ganglia FDG	−0.4413	<0.0001	<0.0001
	Thalamus FDG	−0.4663	<0.0001	<0.0001
	Temporal FDG	−0.3085	0.0024	0.0053
Biomarker	Galectin3	−0.1478	0.327	0.7034
	MMP9	0.0319	0.8333	0.9574
	PDGF‐BB	0.1065	0.4810	0.817
	VCAM1	0.1914	0.2025	0.6376
	VEGFA	0.1708	0.2564	0.703
	TGFβ1	−0.0568	0.7078	0.9574
	CX3CL1	0.1336	0.3760	0.7264
	GFAP	0.4026	0.0055	0.1570
	NFL	0.1317	0.3828	0.7312
	*t*‐tau	0.5019	0.0004	0.0274

Abbreviations: ADL, activities of daily living; bvFTD, behavioral variant frontotemporal dementia; CDR, Clinical Dementia Rating scale; CX3CL1, C‐X3‐C motif chemokine ligand 1; FBI, Frontal Behavior Inventory; FDG, fluorodeoxyglucose F18; FDR, false discovery rate; FTLD, frontotemporal lobar degeneration; GFAP, glial fibrillary acidic protein; GMV, gray matter volume; MMP, matrix metalloproteinase; NACC, National Alzheimer's Coordinating Center; NFL, neurofilament light chain; NPI‐Q,; Neuropsychiatric Inventory Questionnaire; PDGF‐BB, platelet‐derived growth factor‐BB; SOB, sum of boxes; TGF‐β1, transforming growth factor‐beta 1; *t*‐tau, total tau; VCAM1, vascular cell adhesion molecule‐1; VEGFA, vascular endothelial growth factor A.

*Clinical correlations*: Whole‐brain FW was positively correlated with disease severity, as measured by the FTLD‐CDR SOB (*r *= 0.4478, *p *< 0.0001, *p*FDR < 0.0001), CDR sum of box score (*r *= 0.4741, *p *< 0.0001, *p*FDR < 0.0001), and impairments in ADL (*r *= 0.3577, *p *= 0.0001, *p*FDR = 0.0023). It was negatively correlated with MMSE (*r *= −0.3446, *p *= 0.0002, *p*FDR = 0.0030) and MoCA (*r *= −0.3133, *p *= 0.0009, *p*FDR = 0.0077). Furthermore, higher FW was associated with worse neuropsychiatric symptoms, including higher scores of NPI‐Q (*r *= 0.2343, *p *= 0.0142, *p*FDR = 0.0442), FBI total (*r *= 0.2010, *p *= 0.0361, *p*FDR = 0.0862) and disinhibition subscore (*r *= 0.1916, *p *= 0.0460, *p*FDR = 0.0966).

*Neuroimaging correlations*: Strong negative correlations were observed between FW and GMV, with the strongest associations in the basal ganglia (*r *= −0.6247, *p *< 0.0001, *p*FDR < 0.0001), frontal lobe (*r *= −0.5493, *p *< 0.0001, *p*FDR < 0.0001), and thalamus (*r *= −0.4777, *p *< 0.0001, *p*FDR < 0.0001). Similarly, FW showed strong negative correlations with regional glucose metabolism (FDG), most prominently in the frontal lobe (*r *= −0.7080, *p *< 0.0001, *p*FDR < 0.0001), limbic region (*r *= −0.5507, *p *< 0.0001, *p*FDR < 0.0001), and basal ganglia (*r *= −0.4413, *p *< 0.0001, *p*FDR < 0.0001).

*Plasma biomarker correlations*: A positive correlation was found with the astrocytic marker GFAP (*r *= 0.4026, *p *= 0.0055, *p*FDR = 0.1570) and *t*‐tau (*r *= 0.5019, *p *= 0.0004, *p*FDR = 0.0274)

Of note, several variables that did not show significant correlations in whole‐brain FW demonstrated statistically significant associations in tract‐specific analyses. Neuroimaging correlations revealed that temporal GMV was significantly negatively correlated with FW in the left cingulum hippocampus (*r *= −0.5892, *p* < 0.0001, *p*FDR < 0.0001), right cingulum hippocampus (*r *= −0.4332, *p *< 0.0001, *p*FDR < 0.0001), left inferior longitudinal fasciculus (*r *= −0.5853, *p* < 0.0001, *p*FDR < 0.0001), right inferior longitudinal fasciculus (*r *= −0.4624, *p* < 0.0001, *p*FDR < 0.0001), left superior longitudinal fasciculus (*r *= −0.2806, *p* = 0.0031, *p*FDR = 0.0067), left uncinate fasciculus (*r *= −0.2855, *p* = 0.0026, *p*FDR = 0.0058), and right uncinate fasciculus (*r *= −0.3340, *p* = 0.0004, *p*FDR = 0.0011). Biomarker correlations show VCAM1 was positively correlated with FW in the right anterior thalamic radiation (*r *= 0.3694, *p* = 0.0115, *p*FDR = 0.1958). VEGFA was positively correlated with FW in the left corticospinal tract (*r *= 0.3518, *p* = 0.0165, *p*FDR = 0.2339). These correlations did not survive FDR correction.

For clinical correlations, language function showed significant negative correlations with FW in the left anterior thalamic radiation (*r *= −0.2833), left superior longitudinal fasciculus (*r *= −0.2470), and left uncinate fasciculus (*r *= −0.2442) (all *p*FDR < 0.05). FBI total scores demonstrated positive correlations with FW across multiple WM tracts, with the strongest associations in the left inferior longitudinal fasciculus (*r *= 0.2791) and left cingulum hippocampus (*r *= 0.2405) (both *p*FDR < 0.05). FBI apathy scores were positively correlated with FW in the right anterior thalamic radiation (*r *= 0.2520) and left inferior longitudinal fasciculus (*r *= 0.2631) (both *p*FDR < 0.05). FBI disinhibition scores showed positive correlations with FW in the left uncinate fasciculus (*r *= 0.247) and left inferior longitudinal fasciculus (*r *= 0.2368) (both *p*FDR < 0.05). Behavioral symptoms were positively associated with FW in right anterior thalamic radiations (*r *= 0.2448, *p*FDR < 0.05).

### 3.5 Brain changes mediate the association between FW and clinical scales

Mediation analyses were conducted to examine whether the associations between whole‐brain FW and clinical scores were mediated by brain changes. Significant mediation effects (ACME, *p* < 0.05) were observed across multiple brain regions, as shown in Table 4.

**TABLE 4:** Mediation analysis results of free water on clinical scores via neuroimaging variables.

Mediator	Y	ACME	*P*	ADE	*P*	Total effect	*P*	Proportion
Frontal GMV	ADL	43.49	0.0022	52.22	0.0562	95.71	<0.0001	0.4521
	NPI‐Q	52.15	0.0052	24.03	0.5052	76.18	0.0164	0.6783
Limbic GMV	ADL	25.84	0.0474	70.01	0.0082	95.85	0.0002	0.2671
	NPI‐Q	53.09	0.0022	22.40	0.5224	75.49	0.0156	0.6953
Occipital GMV	MMSE	−10.08	0.0200	−39.32	0.0016	−49.39	0.0002	0.1982
	ADL	25.91	0.0028	69.75	0.0034	95.66	<0.0001	0.2658
	NPI‐Q	22.82	0.0430	52.50	0.1154	75.31	0.0166	0.2940
Parietal GMV	MMSE	−11.59	0.0134	−37.99	0.0034	−49.58	0.0002	0.2284
	ADL	29.63	0.0004	66.58	0.0064	96.20	<0.0001	0.3031
	NPI‐Q	29.93	0.0114	45.15	0.1632	75.08	0.0138	0.3921
Basal ganglia GMV	CDR® plus NACC FTLD‐SOB	12.50	0.0264	30.05	0.0016	42.56	<0.0001	0.2933
	CDR SOB	9.79	0.0456	30.95	0.0002	40.75	<0.0001	0.2396
	ADL	40.39	0.0162	55.60	0.0580	95.98	<0.0001	0.4164
	NPI‐Q	50.60	0.0252	24.69	0.5230	75.29	0.0214	0.6590
Thalamus GMV	CDR® plus NACC FTLD‐SOB	9.40	0.0182	33.16	0.0004	42.56	<0.0001	0.2158
	ADL	33.28	0.0054	62.94	0.0160	96.23	<0.0001	0.3403
Frontal FDG	ADL	58.08	0.0268	81.75	0.0320	139.83	<0.0001	0.4137

Abbreviations: ACME, average causal mediation effect; ADE, average direct effect; ADL, activities of daily living scale; CDR SOB, the sum of the box scores of the six domains of the CDR; CDR plus NACC FTLD‐SOB, sum of boxes scores of the six domains of the CDR plus the behavior and language domains; FDG, fluorodeoxyglucose uptake; GMV, gray matter volume; MMSE, Mini‐Mental State Examination; NPI‐Q, Neuropsychiatric Inventory Questionnaire.

Frontal GMV significantly mediated the effects of FW on both the ADL (ACME = 43.49, *p* = 0.0022) and the NPI‐Q (ACME = 52.15, *p* = 0.0052), accounting for 45.21% and 67.83% of the total effects, respectively. The direct effect on ADL was marginally significant (ADE = 52.22, *p* = 0.0562), while that on NPI‐Q was not significant (ADE = 24.03, *p* = 0.5052). Total effects were significant for both (*p* < 0.0001; *p* = 0.0164). Frontal FDG mediated the association between FW and ADL (ACME = 58.08, *p *= 0.0268; 41.37% mediated), with both direct (*p* = 0.0320) and total effects (*p* < 0.0001) being significant.

Limbic GMV also showed significant mediation for the association between FW and ADL (ACME = 25.84, *p* = 0.0474; 26.71% mediated) and NPI‐Q (ACME = 53.09, *p* = 0.0022; 69.53% mediated). The direct effect is significant for ADL (ADE = 70.01, *p* = 0.0082) but not for NPI‐Q (ADE = 22.40, *p* = 0.5224).

Occipital GMV mediated FW relationships with MMSE (ACME = −10.08, *p* = 0.0200), ADL (ACME = 25.91, *p* = 0.0028), and NPI‐Q (ACME = 22.82, *p* = 0.0430). Direct effects were significant for MMSE and ADL but not NPI‐Q.

Parietal GMV significantly mediated the association between FW and MMSE (ACME = −11.59, *p* = 0.0134), ADL (ACME = 29.63, *p* = 0.0004), and NPI‐Q (ACME = 29.93, *p* = 0.0114). Direct effects were significant for MMSE and ADL only. Basal ganglia GMV mediated CDR® plus NACC FTLD‐SOB (ACME = 12.50, *p* = 0.0264), CDR SOB (ACME = 9.79, *p* = 0.0456), ADL (ACME = 40.39, *p* = 0.0162), and NPI‐Q (ACME = 50.60, *p* = 0.0252). Direct effects were non‐significant for ADL and NPI‐Q. Thalamus GMV significantly mediated CDR® plus NACC FTLD‐SOB (ACME = 9.40, *p* = 0.0182) and ADL (ACME = 33.28, *p *= 0.0054), with significant direct effects for both.

## 4 DISCUSSION

This study is the first systematic investigation of the correlations between FW alterations and other multimodal biomarkers in patients with bvFTD. It provides a comprehensive analysis of the relationships between FW changes and other glymphatic system markers – such as PVS and the ALPS index – as well as clinical symptoms, neuroimaging characteristics, and plasma biomarkers. By integrating multi‐dimensional evidence, this study significantly advances the understanding of how FW dynamics contribute to the pathology of bvFTD. Moreover, through mediation analyses, the study elucidates potential pathological pathways, demonstrating how FW mediates the relationship between PVS and ALPS and how changes in FW associated with GM atrophy and hypometabolism may drive clinical manifestations. These insights enhance the current knowledge of glymphatic system impairment in bvFTD and highlight the central role of FW in disease mechanisms.

### 4.1 Alterations of FW in bvFTD patients

This study confirms that, compared to the normal control group, patients with bvFTD exhibit significantly elevated FW levels in both the whole brain and multiple regional WM tracts. The most pronounced differences were observed in the uncinate fasciculus and the cingulum hippocampus, which connect the frontal and temporal lobes. Axonal damage, myelin breakdown, and expansion of the extracellular space – all hallmarks of neurodegenerative changes – can lead to the accumulation of extracellular fluid, manifesting as increased FW.^6^ This is consistent with previous studies reporting FW alteration in FTD.^19^, ^22^ Previous research also showed that FW was elevated in AD, where it correlated with clinical features (such as episodic memory and executive function) and pathological protein deposition (amyloid beta [Aβ] or tau),^9^, ^10^, ^11^, ^12^, ^13^, ^14^, ^15^, ^16^, ^17^, ^23^, ^24^ as well as plasma P‐TAU217,^18^ GFAP, and NFL.^19^ Elevated FW has also been reported in mild cognitive impairment (MCI),^25^, ^26^ and even in the subjective cognitive decline (SCD) stage.^27^ One study indicated that FW levels in the hippocampus of early MCI patients were already higher than in controls. At the same time, hippocampal volume showed no significant difference at that stage, demonstrating the sensitivity of FW as a biomarker in early disease phases.^17^ Furthermore, elevated FW has also been detected in DLB^20^, ^28^, ^29^ and in cerebral small vessel disease‐related vascular dementia.^30^, ^31^ Therefore, elevated FW is not disease‐specific but represents a common pathway across multiple neurodegenerative disorders.

### 4.2 Association between FW levels and clinical features

In terms of clinical correlations, FW levels showed positive correlations with the CDR® plus NACC FTLD‐SOB score, the CDR total score, and the ADL score, while exhibiting negative correlations with the MMSE and MoCA scores. Additionally, FW levels were positively correlated with NPI‐Q and FBI score. These findings indicate that elevated FW levels parallel the progression of dementia severity in bvFTD patients. Tract‐specific analyses further elucidated the anatomical basis of these associations. This study found that FW levels in the left uncinate fasciculus were most significantly correlated with NPI‐Q scores, which can be attributed to the role of the left uncinate fasciculus as a key pathway connecting the left frontal lobe (a core hub for behavioral regulation) and the left temporal pole (a critical region for social cognition and emotional processing).^32^

### 4.3 Association between FW levels and neuroimaging markers

FW was significantly associated with PVS enlargement and ALPS, and further mediation analysis confirmed that FW fully mediated the effect of PVS on ALPS, with no significant direct pathway. This confirmed that PVS was closely associated with increased FW accumulation in the brain, which in turn could impair glymphatic clearance function. Previous research also explored the relationship between FW and other glymphatic indicators. One study^12^ found that FW fraction in the choroid plexus of Aβ‐positive participants was associated with a reduced DTI‐ALPS index. A study across four independent middle‐aged and elderly cohorts more definitively demonstrated that WM‐FW mediated the association between ALPS and executive function.^30^ In our previous studies, we demonstrated alterations in the DTI‐ALPS index and their associations with clinical, imaging, and blood biomarkers,^2^, ^5^ and this study further extends the understanding of glymphatic system function to the correlation between PVS, WM‐FW, and ALPS.

Additionally, FW levels exhibited strong negative correlations with GMV and FDG metabolism in multiple brain regions. The strongest negative correlations with GMV were observed in the basal ganglia, frontal lobe, and thalamus, while the strongest negative correlations with FDG were found in the frontal lobe, limbic system, and basal ganglia. These regions are core areas involved in cognitive, emotional, and motor functions, and their atrophy and metabolic decline are characteristic neuroimaging features of bvFTD.^33^ The close associations between FW and these neuroimaging markers suggest that WM microstructural damage in bvFTD is closely linked to GM atrophy and reduced cerebral metabolism, collectively driving disease progression.

### 4.4 Association between FW levels and plasma biomarkers

In plasma biomarker studies, GFAP reflects neuroinflammatory processes in FTD.^34^, ^35^ Total‐tau (*t*‐tau) and NFL are indicative of neuronal injury.^35^ We selected MMP‐9, PDGF‐BB, TGF‐β1, galectin‐3, and VCAM‐1 as biomarkers because they reflect critical aspects of vascular dysfunction, including remodeling, endothelial activation, and blood–brain barrier disruption.^36^, ^37^, ^38^, ^39^, ^40^ This selection was validated by our recent work, in which we comprehensively characterized these vascular markers in a bvFTD cohort. Specifically, we observed that MMP‐9 and VCAM1 levels were significantly elevated, while PDGF‐BB levels were significantly reduced, with no significant differences in galectin‐3.^5^ We found that FW showed a positive correlation with plasma GFAP and *t*‐tau, suggesting that elevated WM‐FW in bvFTD is closely linked to neuroinflammation and axonal injury. The strong association between FW and neuroinflammation is supported by previous research, which reported that FW was more strongly correlated with GFAP than with NfL in neurodegenerative disease.^19^ However, no significant correlations were observed between FW and other biomarkers in whole‐brain analyses. In tract‐specific analyses, only VCAM1 and VEGFA showed positive correlations with FW in specific fiber tracts. This may be due to the relatively small sample size in biomarker subgroups, necessitating further validation in larger cohorts. In addition, previous studies demonstrated that placental growth factor may contribute to cognitive impairment through the accumulation of WM‐FW,^41^ which could be examined in our future cohort studies.

### 4.5 Mediating role of neuroimaging markers between FW and clinical scores

Mediation analysis further revealed that GMV (in the frontal, limbic, occipital, parietal, basal ganglia, and thalamus) and frontal FDG metabolism mediated the association between FW and clinical scores in bvFTD patients. These results indicate that the impact of elevated FW on clinical symptoms in bvFTD is achieved through reductions in GMV and decreased cerebral glucose metabolism. Regarding the mediating effects related to core behavioral symptoms, the mediating effect of frontal lobe GMV on the relationship between FW and NPI‐Q scores was the most prominent, with a mediation proportion as high as 67.83%, and the direct effect was not significant. This suggests that the pathological process of bvFTD involves a complex network of WM‐FW changes, GM atrophy, and metabolic decline. Additionally, frontal lobe FDG metabolism mediated the relationship between FW and ADL scores with a proportion of 41.37%, and both the direct and total effects were significant. This result further complements the mediation mechanism from a brain function perspective. The dominant mediation by frontal and limbic GMV underscores the critical role of frontal‐striatal circuits, suggesting that WM pathology drives downstream frontal atrophy and consequent behavioral deficits.^42^, ^43^

### 4.6 Limitations of this study

Nevertheless, this study has several limitations. First, as a cross‐sectional design, it cannot establish a causal relationship between FW and the progression of bvFTD, and the mediation results should be interpreted cautiously. Longitudinal studies are required to monitor dynamic changes in FW and its predictive value for clinical outcomes. Second, although multiple biomarkers were analyzed, the subgroup with both FW metrics and plasma biomarker data was relatively small (*n* = 49), which may lead to potential selection bias and reduce the statistical power and affect the robustness of the correlation analyses. Future studies with larger sample sizes, possibly through multi‐center collaborations, are needed to validate these associations and increase statistical power, and potential associations (such as specific fiber tracts and VCAM1/VEGFA) merit further exploration in expanded cohorts. Third, previous studies identified influences of factors such as age, sex, and genetic variants (e.g., *TMEM106B, PTK2B*, *WNT3*, and *APOE*) on FW or WM microstructure^44^, ^45^, ^46^, ^47^; however, these were not addressed in the current study and should be investigated in future large‐sample research. Fourth, while our study benefited from a large sample size collected over years using a single scanner, we acknowledge that subtle temporal variations or scanner drift over time could influence the imaging measurements.

Finally, the reliability of our FW estimates was constrained using single‐shell data, which lacks the specificity of multi‐shell acquisitions. As recent validation studies highlight, single‐shell fitting cannot definitively distinguish true increases in extracellular fluid from alterations in intrinsic tissue diffusivity.^48^ Consequently, our FW measures should be interpreted as a composite signal reflecting both fluid and microstructural changes. Future multi‐shell studies are warranted to disentangle these compartments with higher specificity.

## 5 CONCLUSION

This study is the first to identify significantly elevated FW levels in global and specific WM tracts in bvFTD patients and confirmed its close association with PVS and ALPS, clinical severity, brain structure, cerebral metabolism, and plasma biomarkers. Additionally, FW mediates the association between PVSs and ALPS, and brain atrophy and metabolism mediate the relationship between FW and clinical scores. These findings indicate that extracellular FW accumulation serves a critical role in the pathophysiology of bvFTD, contributing to the glymphatic system, clinical severity, brain changes, and inflammation.

## CONFLICT OF INTEREST STATEMENT

The authors declare no conflicts of interest. Author disclosures are available in the Supporting Information.

## CONSENT STATEMENT

This study was conducted following the ethical standards laid out in the 1974 Declaration of Helsinki and its later amendments and approved by the Ethics Committee of Xuanwu Hospital, Capital Medical University. Written informed consent was obtained from all participants and their families.

## Supporting information



Supporting Information
